# Intra-tumoural lipid composition and lymphovascular invasion in breast cancer via non-invasive magnetic resonance spectroscopy

**DOI:** 10.1007/s00330-020-07502-4

**Published:** 2020-12-03

**Authors:** Sai Man Cheung, Ehab Husain, Vasiliki Mallikourti, Yazan Masannat, Steven Heys, Jiabao He

**Affiliations:** 1 Institute of Medical Sciences, School of Medicine, University of Aberdeen, Foresterhill, Aberdeen, AB25 2ZD UK; 2 Pathology Department, Aberdeen Royal Infirmary, Aberdeen, UK; 3 Breast Unit, Aberdeen Royal Infirmary, Aberdeen, UK

**Keywords:** Monounsaturated fatty acids (MUFA), Triglycerides, Saturated fatty acids (SFA), Serotonin, Magnetic resonance spectroscopy (MRS)

## Abstract

**Objectives:**

Despite improved survival due to new treatments, the 10-year survival rate in patients with breast cancer is approximately 75%. Lymphovascular invasion (LVI), a prognostic marker independent from histological grade and stage, can only be fully determined at final histological examination. Lipid composition is deregulated in tumour via de novo lipogenesis, with alteration in lipogenic genes in LVI. We hypothesise alteration in lipid composition derived from novel non-invasive spectroscopy method is associated with LVI positivity.

**Methods:**

Thirty female patients (age 39–78) with invasive ductal carcinoma were enrolled, with 13 LVI negative and 17 LVI positive. Saturated, monounsaturated, polyunsaturated fatty acids and triglycerides (SFA, MUFA, PUFA and TRG) were quantified from ex vivo breast tumours freshly excised from patients on a 3 T clinical MRI scanner, and proliferative activity marker Ki-67 and serotonin derived histologically.

**Results:**

There were significantly lower MUFA (*p* = 0.0189) in LVI positive (median: 0.37, interquartile range (IQR): 0.25–0.64) than negative (0.63, 0.49–0.96). There were significantly lower TRG (*p* = 0.0226) in LVI positive (1.32, 0.95–2.43) than negative (2.5, 1.92–4.15). There was no significant difference in SFA (*p* = 0.6009) or PUFA (*p* = 0.1641). There was no significant correlation between lipid composition against Ki-67 or serotonin, apart from a borderline negative correlation between PUFA and serotonin (*r* = - 0.3616, *p* = 0.0496).

**Conclusion:**

Lipid composition might provide a biomarker to study lymphovascular invasion in breast cancer.

**Key Points:**

*• Monounsaturated fatty acids in lymphovascular invasion (LVI) positive invasive breast carcinoma were significantly lower than that in LVI negative.*

*• Triglycerides in LVI positive invasive breast carcinoma were significantly lower than that in LVI negative.*

*• Lipid composition from MR spectroscopy reflects the rate of de novo lipogenesis and provides a potential biomarker independent from histological grade and stage.*

**Supplementary Information:**

The online version contains supplementary material available at 10.1007/s00330-020-07502-4.

## Introduction

The 10-year survival rate in patients with breast cancer is approximately 75% [1], despite the improved survival [1] as a result of early detection, screening and new treatments [2]. Lymphovascular invasion (LVI) is an important histopathological feature of prognostic value independent from histological grade [3] that correlates with proliferative activity marker Ki-67 [4] and nodal status [5]. However, the full assessment of LVI is achieved at the final histology [6] due to partial sampling error in pre-operative biopsy [7]. Other factors including tissue shrinkage (although immunohistochemistry might be used to highlight endothelial cells) [8] and mechanical force–induced cell displacement [9] can affect the assessment, especially after tumour dissipation induced by neoadjuvant chemotherapy can make histological assessment of LVI difficult. LVI is associated with elevated risk of lymph node metastasis [10] through tumour-associated lymphangiogenesis [11], and the expression alterations of genes in the incorporation of saturated- and monounsaturated fatty acids (SFA, MUFA) in phospholipid metabolism [12]. Pro-inflammatory polyunsaturated fatty acid (PUFA)–derived eicosanoids recruit macrophages to trigger immune response leading to elevated serotonin (5-HT) [13] that is associated with worse 10-year survival [14]. Hence, non-invasive quantification of lipid composition as a marker of LVI has a potential role in personalised care, especially in the context of neoadjuvant treatment.

Lipid composition can be measured using biochemical extraction methods for gas chromatography [15], but suffering from invasiveness, labour-intensive procedures and high cost [16]. Magnetic resonance spectroscopy (MRS) is a powerful tool for non-invasive biochemical quantification with scanners readily available in tertiary hospitals, but unable to discern lipid constituents due to overlapping signals on a one-dimensional spectrum [17]. Localised correlation spectroscopy (L-COSY) is capable of discerning lipid constituents in breast cancer on a two-dimensional spectral map [18], but suffering from water contamination signal and limited accuracy resulted from dispersed peak pattern. Double quantum filtering (DQF), utilising signal selection targeting specific double bonds in molecular structure [19], effectively suppresses contamination signal and creates focused spectral peak. Hence, DQF-MRS can single out PUFA for high accuracy quantification [17], necessary in tumour due to low PUFA fraction, while DQF-COSY provides accurate quantification of lipid composition [19].

We therefore hypothesise there is a difference in lipid composition derived from DQF-MRS and DQF-COSY between LVI status, and is independent from cell division and inflammation in breast cancer.

## Materials and methods

**Fig. 1:**
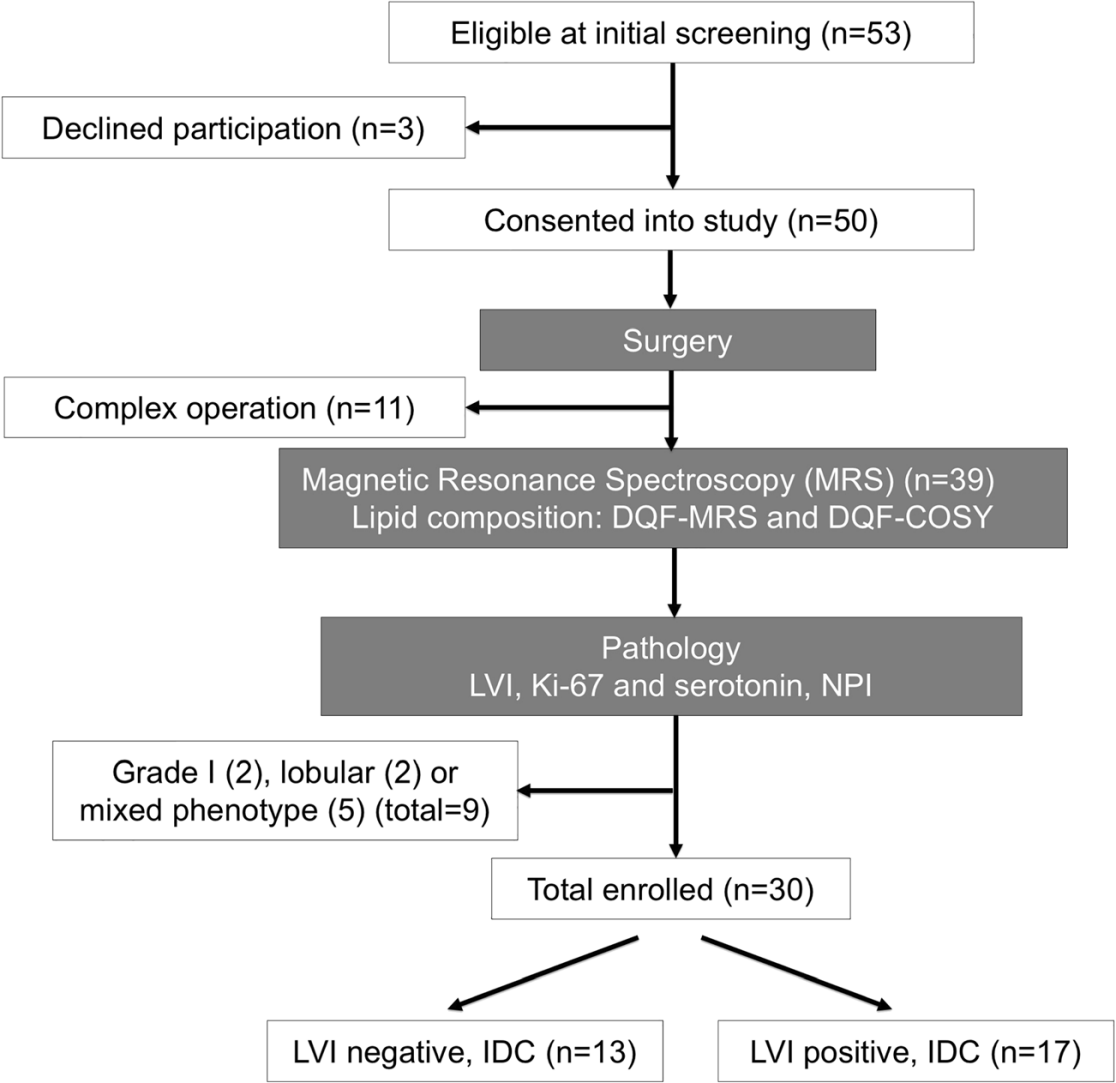
Study design. A two-group cross-sectional study is shown in a flow chart. Fifty-three female breast cancer patients were found to be eligible at initial screening. Upon approach, three patients declined participation and the rest were consented to the study. After wide local excision or mastectomy, the freshly excised tumour specimen was scanned on a 3 T clinical MRI scanner to derive lipid composition using double quantum–filtered (DQF)-MRS and DQF-correlation spectroscopy (DQF-COSY). Immunohistochemical examinations were conducted to assess lymphovascular invasion (LVI), Ki-67 and serotonin expression and Nottingham Prognostic Index (NPI). In total, 30 patients with invasive ductal carcinoma (IDC), 13 with LVI negative and 17 with LVI positive, participated in the study

We conducted a two-group cross-sectional study to quantify lipid composition in whole tumours freshly excised from patients with breast cancer, with further comparison against histopathological findings (Fig. 1).

### Study approval

The study was approved by the North West - Greater Manchester East Research Ethics Committee (REC Reference: 16/NW/0032), and signed written informed consent was obtained from the patients prior to entry into the study.

### Clinical procedure

Thirty female patients (age 39–78) with grade II or III invasive ductal carcinoma participated in the study. Patients over the age of 18, undergoing a wide local excision or mastectomy, and with a tumour size larger than 1 cm in diameter on mammography were eligible. Patients with previous breast malignancies or patients who had neoadjuvant chemotherapy or neoadjuvant hormonal therapy prior to surgery were excluded. In total, 50 patients consented to be included in the study from 53 consecutive patients in the Breast Unit at Aberdeen Royal Infirmary. However, from these 50 resected tumour specimens, 11 were not scanned due to theatre delays and MRI scanner unavailability. A further 9 tumour specimens were excluded due to lobular phenotype (*n* = 2), mixed phenotype (*n* = 5) or grade I (*n* = 2) on final histopathological examination of the excised tumour. Standard histopathological examination was performed to determine tumour size, grade, LVI, lymph node metastasis, receptors and Nottingham Prognostic Index (NPI) [20]. Upon full histological examination, there were 13 LVI negative and 17 LVI positive breast cancers. Immunostaining was conducted in a single batch for Ki-67 (Clone MIB-1, Dako, Dilution 1/100) and serotonin (Clone HTR1A, Thermo Fisher Scientific, Dilution 1/500) including appropriate positive controls, with immunostainer Ventana Benchmark XT with Ultraview DAB Detection Kit (760-500) (Ventana Medical Systems, Inc.), and assessed by a consultant pathologist. Ki-67 was quantitatively assessed as percentage of positive nuclei (0–100%) [21], while serotonin was semi-quantitatively assessed using the H-score: Membranous staining was scored as 0 for ‘no staining’, 1 + for ‘weak staining’, 2 + for ‘moderate staining’ and 3 + for ‘strong staining’. The percentage of cells at different staining intensities was determined by visual assessment. The final score was calculated using the formula 1 × (% of 1 + cells) + 2 × (% of 2 + cells) + 3 × (% of 3 + cells) [22].

### Lipid composition

The freshly excised tumour specimen without formalin treatment was immediately transported to the Aberdeen Biomedical Imaging Centre. The lipid composition spectra were acquired from a single voxel snug fit to the tumour on a 3 T whole-body clinical MRI scanner (Achieva TX, Philips Healthcare) using a 32-channel receiver coil for high sensitivity detection and a body coil for uniform transmission. PUFA spectrum was acquired using DQF-MRS sequence [17] with a repetition time (TR) of 1250 ms, echo time (TE) of 130 ms, spectral editing frequency at 2.8 ppm, bandwidth of 2000 Hz and 1024 points. Reference spectrum (conventional MRS) without water suppression was acquired using PRESS sequence [23] with TR/TE of 1250/130 ms. Lipid spectral map was acquired using DQF-COSY sequence [19], with TR of 552 ms, initial TE of 25 ms, a *t*_*1*_ increment of 1 ms, 256 increments, bandwidth of 1000 Hz and 256 points. The acquisition durations were 10 min 40 s for DQF-MRS and 9 min 45 s for DQF-COSY. Further details of the methodology can be found in the Supplementary Material.

DQF-MRS and DQF-COSY are complementary methods for lipid composition quantification, with respective strength. DQF-MRS targets a single metabolite through the manipulation of quantum states of coupled spins, with the resonance frequency at 2.8 ppm and 5.3 ppm for PUFA [17]. The employment of the field gradient effectively eliminates background signals beyond target metabolite, while the binary nature of quantum coherence pathway selection leads to a 50% loss of the signal [17]. DQF-COSY and L-COSY are localised derivatives from COSY for 2D spectroscopy to allow the identification of molecular structures. L-COSY, without DQF, suffers from overwhelming background signal and broad spectral peak appearance [19]. DQF-COSY embeds DQF component within COSY, allowing suppression of uncoupled signal across the spectral domain and a sharp spectral peak appearance. However, DQF-COSY only retains 25% of the signal, as a consequence of the quantum coherence pathway selection and the use of stimulated echo [19].

PUFA was quantified using DQF-MRS and conventional MRS, demanding minimal discrepancies between the two acquisitions for accuracy. Both acquisitions adopted PRESS sequence backbone [17, 23] with identical voxel localisation RF pulse shape and field gradient strength for identical voxel location, voxel profile and chemical shift displacement. Both acquisitions also adopted identical receiver gain configuration with a shared scanner setup procedure for comparable nominal values and minimal hardware variability.

Lipid constituents were calculated as a ratio of the corresponding spectral peak against the methyl proton peak, without the quantification of absolute concentration using estimated T_1_ and T_2_. PUFA, quantified as spectral peak amplitude at 5.3 pm from DQF-MRS, was referenced to 0.9 ppm from conventional MRS, while all the other lipid constituents, quantified as corresponding spectral peak volumes from DQF-COSY, were referenced to (0.9, 0.9) ppm from DQF-COSY. The PUFA peak at 5.3 ppm from DQF-MRS is spectrally edited through coupling peak at 2.8 ppm [17] equivalent to the cross peak at (5.3, 2.8) ppm in DQF-COSY or 2.8 ppm in conventional 1D MRS, with the ratio against methyl proton proportional to PUFA fraction [24]. The unsaturated fatty acids (UFA) at (2.1, 2.1) ppm from DQF-COSY encompasses PUFA and MUFA substrates manifested at (5.3, 2.8) ppm and (5.3, 2.1) ppm respectively, with the ratio of UFA against methyl proton negatively proportional to SFA fraction (SFA = 1-UFA/methyl) [25, 26].

The peak amplitudes of PUFA (5.3 ppm) and methyl proton (0.9 ppm) were quantified using prior knowledge databases [27] and AMARES algorithm [28] in the jMRUI software (v3.0, TRANSACT) [29]. The peak volumes of UFA, triglycerides (TRG) and MUFA were quantified at (2.1, 2.1) ppm, (4.3, 4.3) ppm and (5.3, 2.1) ppm respectively. Lipid spectral maps were quantified in the Felix software (v2007, Accelrys Inc.), with zerofilling to 512 points and two-dimensional sine bell apodisation [19].

### Statistical analysis

All statistical analysis was performed in the SPSS software (Release 23.0, SPSS Inc.). Normality was determined on all the collected data using the Shapiro-Wilk test. Mann-Whitney *U* tests were used for MUFA, SFA and TRG while independent sample *t* test for PUFA (normally distributed) between LVI groups. Fisher’s exact tests were applied for categorical variables (clinicopathological features). Spearman’s correlation tests were performed between lipid composition against proliferative activity marker Ki-67 and serotonin, while Pearson’s correlation test between PUFA and serotonin in the entire cohort. A *p* value < 0.05 was accepted as being statistically significant.

## Results

The patient demographics are summarised in Table 1. There were no significant differences in age and body mass index (BMI) between groups. There were no significant differences in tumour size, NPI or stage between groups.

**Table 1:** Clinical characteristics of patients. Descriptive statistics of breast cancer patients with histopathological findings are shown for each group and the entire cohort. Numbers are expressed as mean and standard deviation (apart from Nottingham Prognostic Index where median and interquartile range are shown), with pathological entries expressed as number of positive observations

Characteristic	All (*n* = 30)	Lymphovascular invasion (LVI)		*p* value
		Negative (*n* = 13)	Positive (*n* = 17)	
Age (years)	61.1 ± 11.5	59.2 ± 12.6	62.6 ± 10.6	0.4375
Body mass index (BMI)	30.4 ± 6.4	31.2 ± 7.5	29.5 ± 5.4	0.5451
Tumour size (cm)	2.5 ± 0.8	2.4 ± 0.7	2.5 ± 0.8	0.6070
Nottingham Prognostic Index (NPI)	4.41 (3.62–4.56)	4.29 (3.62–4.50)	4.44 (3.70–4.60)	0.4764
*Tumour features*				
pTNM Stage				
I	5	3	2	0.628
II	25	10	15	
Histological grade				
II	15	6	9	1.000
III	15	7	8	
Lymph node involvement	8	2	6	0.407
Oestrogen receptor (ER+)	22	8	14	0.242
Human epidermal growth factor receptor 2 (HER2+)	6	2	4	0.672
Triple-negative breast cancer (TNBC)	7	5	2	0.190

**Fig. 2:**
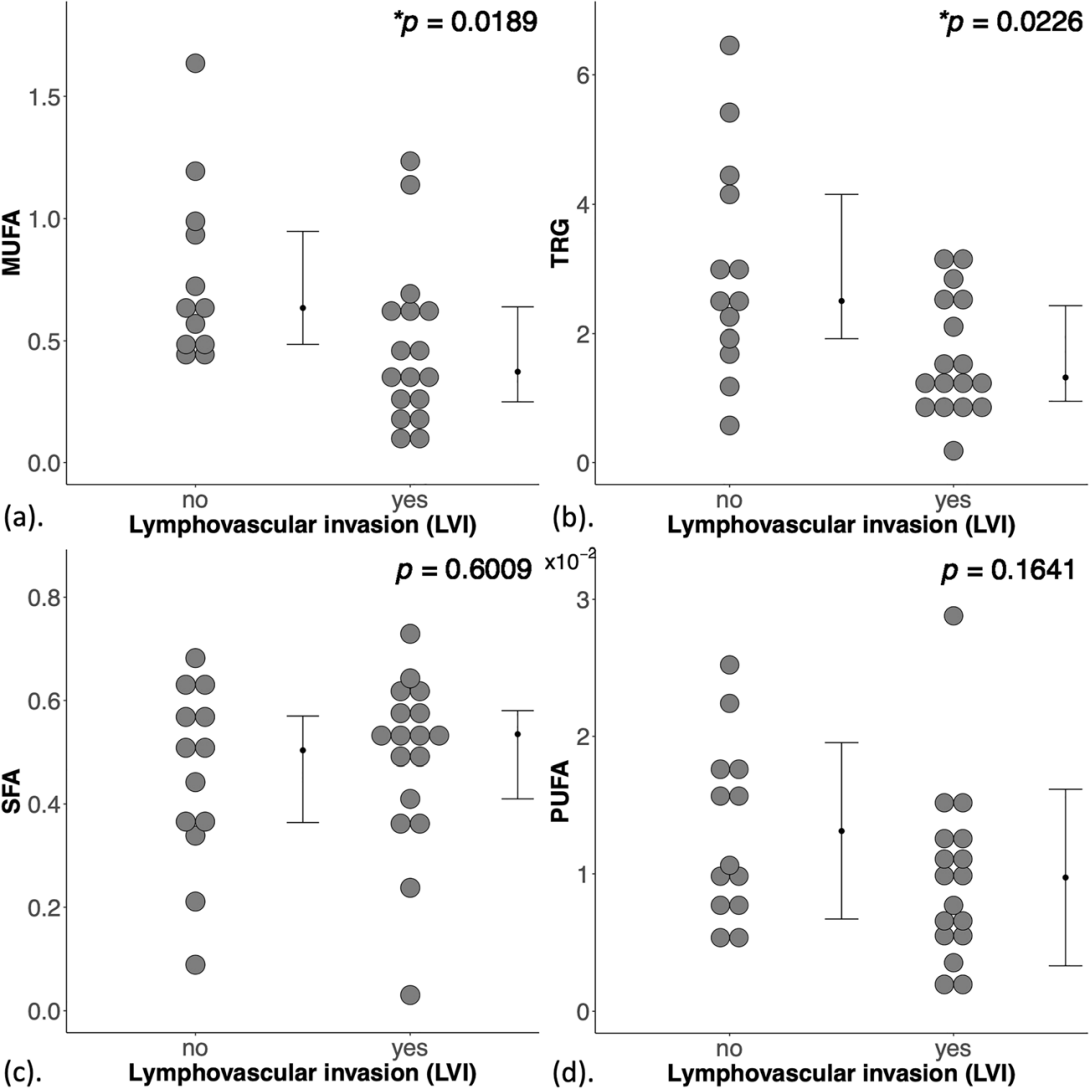
Dot plots to show the group difference in monounsaturated fatty acids (MUFA) (*n* = 12, 17) (**a**), triglycerides (TRG) (*n* = 13, 17) (**b**), saturated FA (SFA) (*n* = 13, 17) (**c**) and polyunsaturated FA (PUFA) (*n* = 13, 17) (**d**) between LVI negative and LVI positive breast tumours. The error bar indicates the median and interquartile range (mean ± SD for PUFA). Mann-Whitney *U* tests (**a**–**c**) and independent sample *t* test (**d**) were performed between the groups and *p* value is shown for each plot. Statistically significant *p* values (< 0.05) are marked by an asterisk

There were significantly lower MUFA (*z* = 2.3470, *p* = 0.0189, Fig. 2a, Table 2) in LVI positive (median: 0.37, interquartile range (IQR): 0.25–0.64) than LVI negative (0.63, 0.49–0.96). There were significantly lower TRG (*z* = 2.2810, *p* = 0.0226, Fig. 2b, Table 2) in LVI positive (1.32, 0.95–2.43) than LVI negative (2.5, 1.92–4.15). There was no significant difference in SFA (*p* = 0.6009, Fig. 2c, Table 2) between LVI negative (0.50, 0.36–0.57) and LVI positive (0.53, 0.41–0.58). There was no significant difference in PUFA (*p* = 0.1641, Fig. 2d, Table 2) between LVI negative (0.013 ± 0.006) and LVI positive (0.010 ± 0.006).

**Table 2:** Lipid composition and histological markers Ki-67 and serotonin in lymphovascular invasion (LVI) negative and positive breast tumours. Correlation scores (Spearman’s *rho* and Pearson’s *r*) of lipid composition, quantified in double quantum–filtered correlation spectroscopy (DQF-COSY) and DQF magnetic resonance spectroscopy (DQF-MRS), against Ki-67 and serotonin are also shown. Monounsaturated, saturated fatty acids (MUFA, SFA) and triglycerides (TRG) were quantified from DQF-COSY, while polyunsaturated FA (PUFA) from DQF-MRS. The spread of lipid composition from DQF-COSY and Ki-67 expression is non-normal and is reported as median and interquartile range (IQR). Significant findings (*p* < 0.05) are marked by ‘*’

LVI group	DQF-COSY			DQF-MRS	Histological markers	
	SFA	MUFA	TRG	PUFA	Ki-67 (%)	Serotonin (0–300)
All (*n* = 30)	0.52 (0.36–0.58)	0.49 (0.37–0.69)	2.01 (1.18–2.99)	0.011 ± 0.007	16.6 (9.7–24.0)	107 ± 62
LVI negative (*n* = 13)	0.50 (0.36–0.57)	0.63 (0.49–0.96)^a^	2.5 (1.92–4.15)	0.013 ± 0.006	17.3 (11.6–29.1)	84 ± 48
LVI positive (*n* = 17)	0.53 (0.41–0.58)	0.37 (0.25–0.64)	1.32 (0.95–2.43)	0.010 ± 0.006	15.3 (9.6–22.9)	124 ± 68
*t/z*-score *p* value	*z* = 0.5230 *p* = 0.6009	*z* = 2.3470 *p* = 0.0189*	*z* = 2.2810 *p* = 0.0226*	*t* = 1.4319 *p* = 0.1641	z = 0.7320 *p* = 0.4639	*t* = 1.9255 *p* = 0.0644
Correlation (*r*/*ρ*-score, *p* value)						
Ki-67	0.2254, 0.2312	0.3108, 0.1008	0.0621, 0.7445	0.2071, 0.2721		
Serotonin	- 0.1444, 0.4465	0.0212, 0.9129	0.1312, 0.4894	- 0.3616, 0.0496*		

^a^MUFA from one LVI negative breast tumour was not quantifiable due to low signal-to-noise ratio, i.e., *n* = 12

**Fig. 3:**
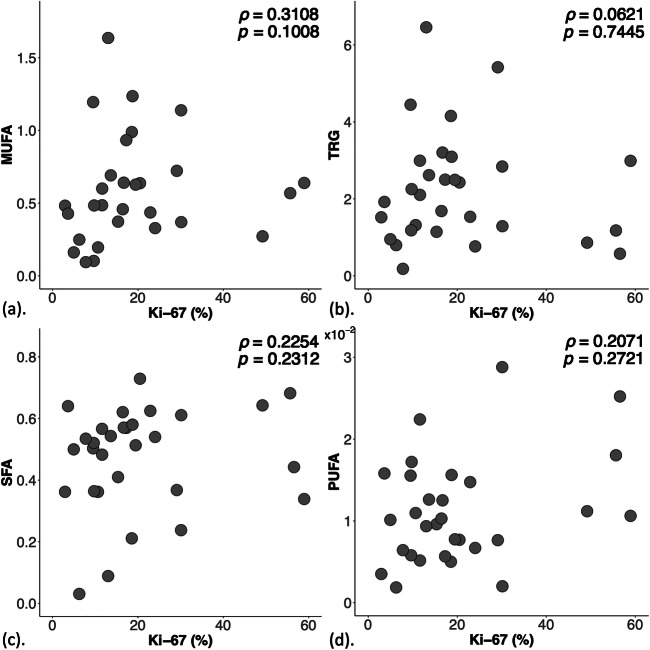
Scatter plots to show the correlation of monounsaturated fatty acids (MUFA) (*n* = 29) (**a**), triglycerides (TRG) (*n* = 30) (**b**), saturated FA (SFA) (*n* = 30) (**c**), and polyunsaturated FA (PUFA) (*n* = 30) (**d**) with Ki-67 expression within the entire cohort. Spearman’s rank correlation tests were performed and *rho* (*ρ*) scores and *p* values are displayed. There were no significant correlations between MUFA, TRG, SFA or PUFA with Ki-67 expression

**Fig. 4:**
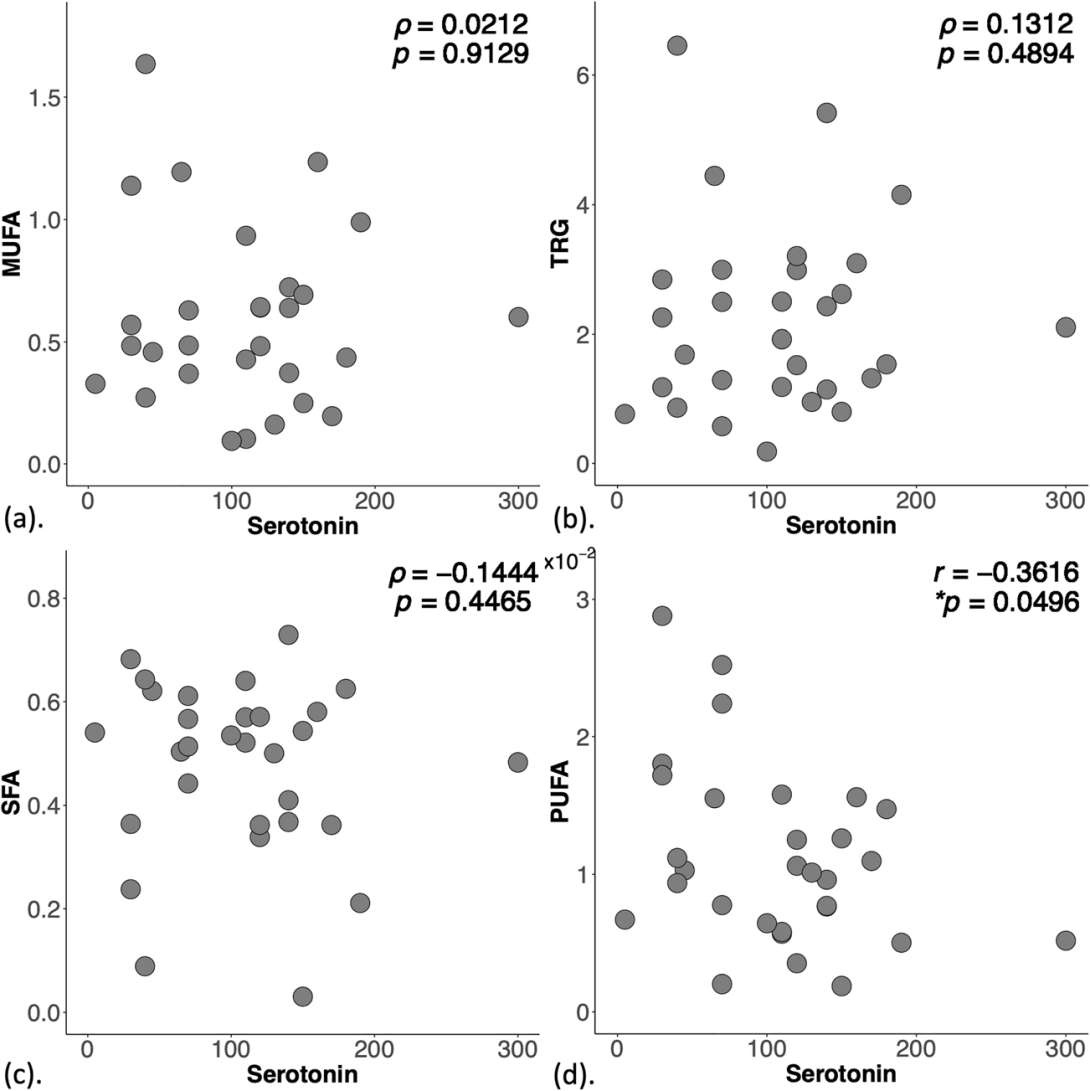
Scatter plots to show the correlation of monounsaturated fatty acids (MUFA) (*n* = 29) (**a**), triglycerides (TRG) (*n* = 30) (**b**), saturated FA (SFA) (*n* = 30) (**c**) and polyunsaturated FA (PUFA) (*n* = 30) (**d**) with serotonin expression within the entire cohort. Spearman’s rank correlation (**a**–**c**) and Pearson’s correlation (**d**) tests were performed and corresponding *rho* (*ρ*) and *r* scores and *p* values are displayed. Statistically significant *p* values (< 0.05) are marked by an asterisk

There were no significant correlations between lipid composition against Ki-67 (Fig. 3, Table 2). There were no significant correlations between MUFA, TRG and SFA against serotonin, but a borderline significant negative correlation between PUFA and serotonin (*r* = - 0.3616, *p* = 0.0496, Fig. 4, Table 2).

## Discussion

In this work, we found positive LVI in breast cancer associated with lower MUFA and triglycerides, but not with SFA or PUFA. Lipid compositions were not correlated with serotonin or proliferative activity marker Ki-67, apart from a borderline negative correlation between PUFA and serotonin. Hence, lipid composition in breast tumour might be a potential marker of LVI.

LVI positivity was negatively associated with MUFA and TRG, but not SFA or PUFA. SFA, the main product of de novo lipogenesis, showed no significant difference between LVI status, indicating potential negative feedback to avoid apoptosis [30] and impaired angiogenesis [31] arising from a further increase in SFA. SFA, an essential building block for cell structural growth, is tightly regulated in the endogenous fatty acid metabolism [32]. Excess SFA palmitate inhibits the mitochondrial phospholipid cardiolipin [30] and suppresses the activity of cathepsin critical for the formation of new endothelial cell-lined blood vessels and consequent cancer proliferation [31]. Hence, the equal spread of SFA distribution in LVI positive and LVI negative might indicate a common protective mechanism against lipotoxicity [30]. The increased MUFA in negative LVI indicates an accelerated conversion from excess SFA for membrane phospholipid synthesis [32] and epidermal growth factor signalling [33], while a reduced MUFA observed in positive LVI due to the export of SFA via tumour cells crossing the lymphatic vasculature [11].

The reduced TRG in positive LVI indicates accelerated de novo lipogenesis unconstrained from lipotoxicity mitigated through SFA clearance via tumour cells crossing the lymphatic vasculature. Triglycerides (TRG), occupying spectral peak at (4.3, 4.3) ppm [18], typically maintain a constant ratio against methyl proton since the ratio of head groups and methyl groups in the TRG chains is fixed. However, TRG is broken down into glycerides and fatty acids in tumours under lipolytic enzyme overexpression [34], with the variation in observation primarily attributed to the glycerides. The spectral peak at (4.3, 4.3) ppm has low SNR in low-fat fraction tumours [35, 36] and the contamination signal from mono- and di-glycerides at 3 T [24, 37] may affect experimental accuracy. PUFA showed no association with LVI, indicating the depletion of naturally low abundant PUFA for membrane synthesis [17] remaining unresolved despite the potential import from peri-tumoural adipocytes in positive LVI. Hence, lipid composition is associated with LVI through the amplification of de novo lipogenesis and could impact on peri-tumoural adipocyte lipid content [38].

Lipid composition did not show a significant correlation against proliferative activity marker Ki-67, indicating no evidence to support a direct link between lipids and tumour cell division within the framework of this work. Lipid composition did not show a significant correlation, apart from a borderline negative correlation in PUFA, against serotonin (5-HT), an epigenetically identified marker of poor 10-year survival rate [14]. Hence, serotonin primarily reflects the upregulation in membrane synthesis [13] (more depleted PUFA [17]) induced by environmental factors, rather than intrinsic aggressiveness or rapid division of the tumour cells.

Lipid composition in the breast has been quantified using MRS in the adipose tissue from both patients and healthy volunteers. Conventional STEAM sequence has been used for the quantification of lipid composition in breast adipose tissue from patients at 3 T [39] and healthy volunteers at 7 T [40]. L-COSY has been used to reveal elevated PUFA in breast adipose tissue in BRCA1 gene mutations carriers compared to healthy controls [18]. Spatially resolved MRS using multiple gradient-echo imaging, an extension from the Dixon method, revealed elevated peri-tumoural SFA and suppressed MUFA in postmenopausal patients compared to healthy controls [41, 42]. However, the investigation of intra-tumoural lipid composition has been limited to fat fraction owing to low abundance of lipids inside breast tumour [43], with the feasibility as a potential marker for response to chemotherapy [35] and the identification of tumour subtypes [36] attempted. The introduction of DQF-MRS allowed the observation of substantially reduced PUFA in breast tumours [17], and this work augmented the understanding of lipid composition in breast cancer [44] using the most sensitive MRS approaches through a formal clinical study on LVI.

This study is the first to investigate the association between lipid composition and LVI positivity in whole human breast tumours. The quantitative nature of the novel spectroscopy method provides an objective approach for LVI evaluation, compared to time-consuming and experience-dependent histological approaches. The quantification at whole tumour estimates an overall degree of compromise in lymphatic vasculature [11], eliminating partial sampling error inherent in biopsy approaches (high false negative of 40% in this study alone) [7]. The ex vivo study design allows the elimination of biological noise and employment for accurate quantification, particularly in the context of depleted PUFA and triglycerides. The deployment of DQF approach suppresses contamination signals proximal to PUFA and triglycerides in the spectral domain, further enhancing quantification accuracy. The feasibility of the employed MRS approaches for in vivo application has been demonstrated using standard clinical scanner, while the clinical utility with underpinning sensitivity to critical tumour metabolic pathways has been shown as an initial step in clinical translation. The lipid composition coupled with current clinical dynamic contrast-enhanced (DCE) and diffusion-weighted MRI may enhance the diagnostic accuracy to reduce false-positive rate.

SFA and TRG are determined from diagonal peaks, with MUFA from cross peak, leading to a difference in scaling from underlying population in energy states. Hence, the comparison is performed on individual lipid constituent across patients, and comparison of nominal values between peaks will require further correction on the scaling factors. The overlap of the distribution in MUFA or TRG between groups may limit the diagnostic value of lipid composition as a biomarker in LVI. This work, as a precursor for consequent in vivo patient studies, attempted to augment the understanding of lipid composition in breast cancer with limited statistical power for multiple comparison correction. Future large cohort in vivo studies, supported by novel spectral analysis approaches [45], are required to confirm the relationship between lipid composition and LVI. Further trials integrating the lipid composition for treatment planning are critical, since neoadjuvant chemotherapy is more likely to be recommended for LVI positive patients [46].

In conclusion, MUFA and triglycerides are reduced in positive LVI resulting from accelerated de novo lipogenesis. Lipid composition observed through novel spectroscopy method might provide a biomarker to study LVI in breast cancer.

## Supplementary information

ESM 1 (DOCX 4467 kb)

## Abbreviations

COSY: Correlation spectroscopy
DQF: Double quantum filtering
IQR: Interquartile range
LVI: Lymphovascular invasion
MRS: Magnetic resonance spectroscopy
MUFA: Monounsaturated fatty acids
NPI: Nottingham prognostic index
ppm: Parts per million
PRESS: Point resolved spectroscopy
PUFA: Polyunsaturated fatty acids
SFA: Saturated fatty acids
TE: Time of echo
TR: Time of repetition
TRG: Triglycerides
