# Supplementary material for: Intra-tumoural lipid composition and lymphovascular invasion in breast cancer via non-invasive magnetic resonance spectroscopy
Source: Eur Radiol. 2020 Dec 3;31(6):3703–11. doi: 10.1007/s00330-020-07502-4 (PMC8128855; doi:10.1007/s00330-020-07502-4)
Supplement: Supplementary file 1 — (DOCX 4467 kb) [file 330_2020_7502_MOESM1_ESM.docx]

# [Supplementary Materials:]

**Experimental details of double quantum filtered-magnetic resonance spectroscopy (DQF-MRS) and double quantum filtered-correlation spectroscopy (DQF-COSY)**

*In vitro and ex vivo:* Lipid composition quantification was conducted in 50/50

corn oil/ water phantom and freshly excised breast tumour. Experiments for corn oil/water phantom were performed in triplicates. Identical DQF-MRS [1] and DQF-COSY [2] acquisition protocol were used in all experiments using a 32-channel receiver coil.

The SNR of polyunsaturated fatty acids (PUFA) from DQF-MRS was computed as the ratio of peak amplitude against the standard deviation of the real spectrum between [-1, -2] ppm [3-5], while the SNR of monounsaturated fatty acids (MUFA), unsaturated fatty acids (UFA) and triglycerides (TRG) from DQF-COSY were calculated as the ratio of corresponding peak volume against the standard deviation of the real spectral map in the square region with diagonal corners at (6.0,0.0) ppm and (7.0,1.0) ppm (Table S1).

Lipid constituents were calculated as ratio of the corresponding spectral peak against the methyl proton peak. PUFA, quantified as spectral peak amplitude at 5.3 pm from DQF-MRS, was referenced to 0.9 ppm from conventional MRS, while all the other lipid constituents, quantified as corresponding spectral peak volumes from DQF-COSY, were referenced to (0.9,0.9) ppm from DQF-COSY. The PUFA peak at 5.3 ppm from DQF-MRS is spectrally edited through coupling peak at 2.8 ppm [1] equivalent to the cross peak at

(5.3,2.8) ppm in DQF-COSY or 2.8 ppm in conventional 1D MRS, with the ratio against methyl proton proportional to PUFA fraction [6]. The UFA at (2.1,2.1) ppm from DQF-COSY encompasses PUFA and MUFA substrates manifested at (5.3,2.8) ppm and (5.3,2.1) ppm respectively, with the ratio of UFA against methyl proton negatively proportional to SFA fraction (SFA=1- UFA/methyl) [7,8].

The conventional MRS and localised COSY (L-COSY) with and without DQF filtering are shown for 50/50 corn oil/water phantom in Figure S1 and Figure S2, with the background signal suppression efficiency estimated for water signal between [4.4, 5.0] ppm. The suppression efficiency of DQF was approximately 4800:1 in phantom for conventional MRS, and 10:1 in phantom for L-COSY. DQF-MRS was only performed on PUFA to cater for the natural low abundance in breast tumour [1]. The PUFA spectrum and lipid composition spectral map from a typical specimen are shown in Figure S3, with PUFA peak highlighted.

*In vivo:* Multi-dimensional NMR, although highly accurate in the quantification

of lipid composition from whole small *ex vivo* specimens, is not feasible in patient studies because of the clinical requirement for intact specimen for histopathological analysis. This work adopted DQF-MRS [1] and DQF-COSY

[2] for localised acquisition of lipid composition from a single voxel within the specimen, as a precursor for consequent *in vivo* patient studies. The pilot PUFA spectrum and lipid composition spectral map from patients with breast cancer, as a natural consequential step of this work, were acquired with the identical protocol using a 16-channel breast coil on 3T whole body clinical

MRI scanner (Figure S4). The robustness of spatial localisation is hence demonstrated through the significantly lower overall lipid signal within the voxel snug fit to the tumour in agreement with conventional MRS supplied by the scanner vendor.

**Table S1. *In vitro*, *ex vivo* and *in vivo* quantification of lipid composition from DQF-MRS and DQF-COSY**

**Experiment Fatty acids^a^ DQF-MRS DQF-COSY**

|  | SNR^b^ | CoV^c^ | SNR | CoV |
| --- | --- | --- | --- | --- |
|  |  | (%) |  | (%) |
| *In vitro*  **Corn oil**  **(50%water)** PUFA | 76.0 ± 5.8 | 7.6 | 155.3 ± 11.5 | 7.4 |
| (n=3) MUFA | - | - | 652.3 ± 29.9 | 4.6 |
| UFA | - | - | 952.0 ± 36.1 | 3.8 |
| TRG | - | - | 940.0 ± 13.2 | 1.4 |
| *Ex vivo* |  |  |  |  |
| **Tumour** |  |  |  |  |
| (n=30) PUFA | 20.8 ± 23.9 | - | 25.5 ± 25.1 | - |
| MUFA | - | - | 57.9 ± 61.4 | - |
| UFA | - | - | 99.6 ± 120.4 | - |
| TRG | - | - | 125.0 ± 99.2 | - |
| *In vivo* |  |  |  |  |
| **Tumour** PUFA | 12.4 ± 8.5 | - | 13.9 ± 6.5 | - |
| (n=5) MUFA | - | - | 36.5 ± 25.9 | - |
| UFA | - | - | 139.4 ± 69.7 | - |
| TRG | - | - | 129.8 ± 69.1 | - |

^a^ SFA = 1 – UFA/methyl.

^b^ In tumours, standard deviations (SD) of the *cohort* are presented, and repeated measurements were not undertaken due to time constraints.

^c^ Coefficient of Variance (CoV) = standard deviation / mean.


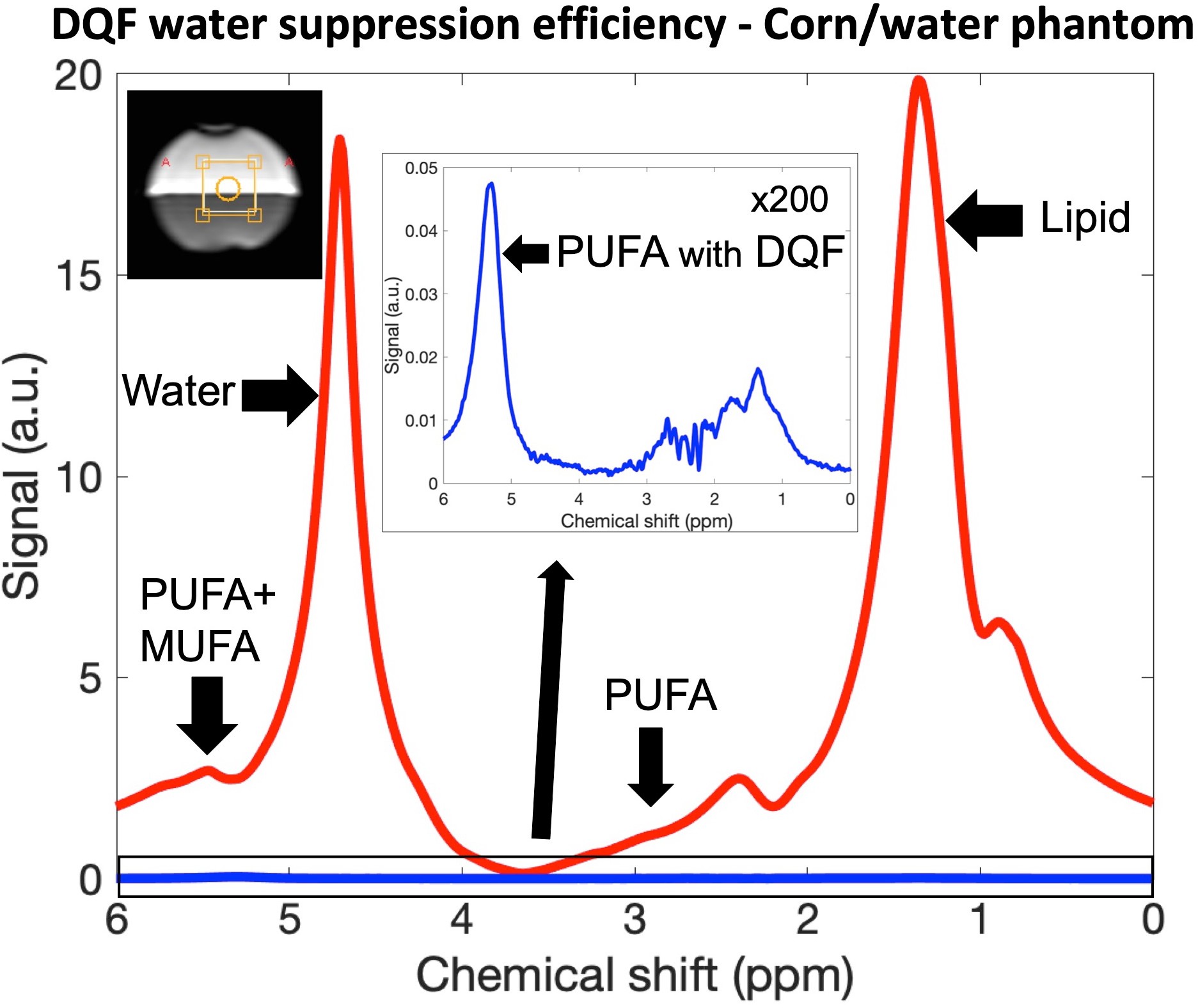


**Figure S1. The lipid detection efficiency (water suppression efficiency) of polyunsaturated fatty acids (PUFA) from double quantum filtered (DQF)-MRS in corn oil/water phantom.**

Reference spectra from volume selection of 50/50 corn oil/water are displayed on the same scale as the DQF-MRS spectra to demonstrate the water suppression efficiency of DQF-MRS. Unsuppressed reference spectrum is shown in red (5.3 ppm from both PUFA and MUFA, 2.8 ppm from PUFA), DQF-MRS PUFA spectrum is shown in blue (5.3 ppm from PUFA). The lipid detection efficiency of DQF-MRS was estimated to be 4800:1 in 50/50 corn oil/water phantom.


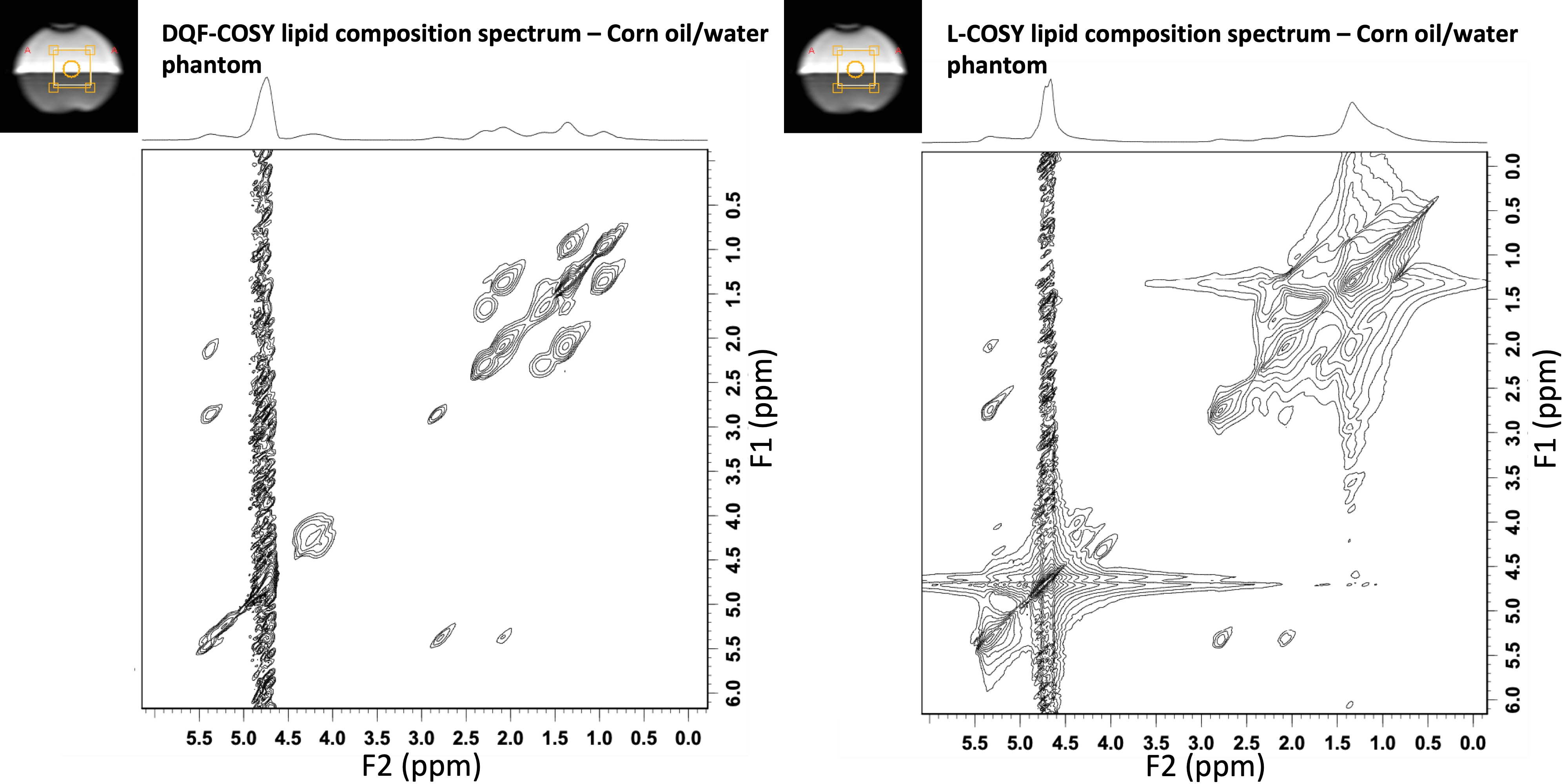


**Figure S2. Double quantum filtered (DQF) – correlation spectroscopy (COSY) and localised (L) – COSY lipid composition spectra from a corn oil/water phantom.**

For DQF-COSY, in volume selection of 50/50 corn oil/water, the major diagonal peaks, including (0.9,0.9) ppm, (2.1,2.1) ppm and (4.3,4.3) ppm are well resolved. Cross peaks at (5.3,2.1) ppm (MUFA) and (5.3,2.8) ppm (PUFA) are prominent with sufficient SNR for quantification. Water resonance at (4.7,4.7) ppm is effectively suppressed. For L-COSY, the contamination from water resonance at (4.7,4.7) ppm is significant. The lipid detection efficiency of DQF-COSY was estimated to be 10:1 in 50/50 corn oil/water phantom.


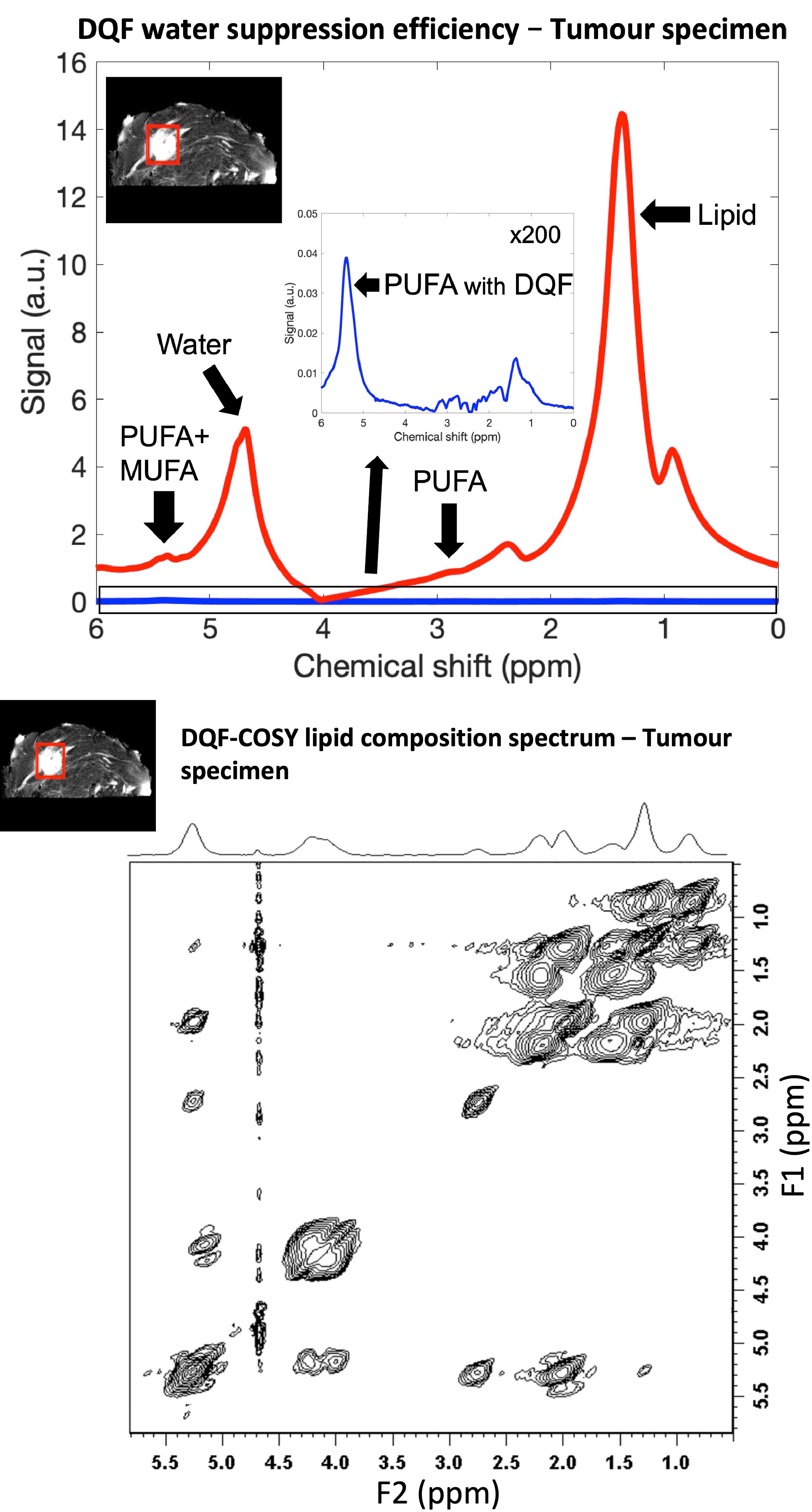


**Figure S3. Reference and polyunsaturated fatty acids (PUFA) double quantum filtered (DQF)-MRS spectra (above) and DQF – correlation spectroscopy (COSY) spectrum (below) from an e*x vivo* breast tumour specimen.**

The volume selection in the surgical specimen is highlighted in red. Unsuppressed reference spectrum is shown in red (5.3 ppm from both PUFA and MUFA, 2.8 ppm from PUFA), DQF- MRS PUFA spectrum is shown in blue (5.3 ppm from PUFA). The DQF-MRS PUFA spectrum is magnified in the inset to show the clean PUFA signal peak for accurate PUFA fraction quantification. Water (4.7 ppm) and lipid resonances (0.0 – 3.0 ppm) are effectively suppressed in the PUFA spectrum. In DQF-COSY, the major diagonal peaks, including (0.9,0.9) ppm, (2.1,2.1) ppm and (4.3,4.3) ppm are well resolved. Cross peaks at (5.3,2.1) ppm (MUFA) and (5.3,2.8) ppm (PUFA) are prominent. The uncluttered spectrum facilitates accurate lipid composition quantification of the tumour.


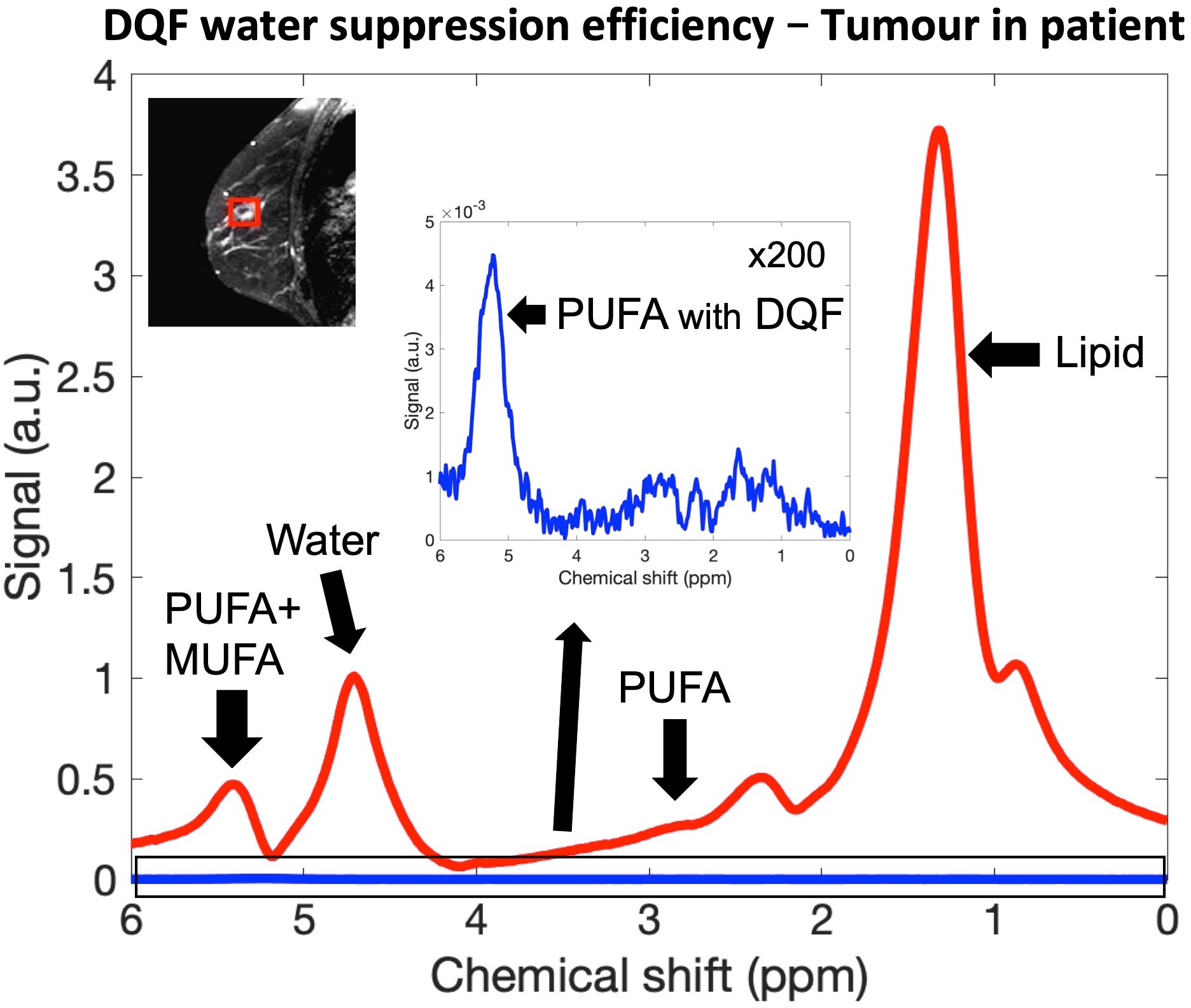


F1 (ppm)


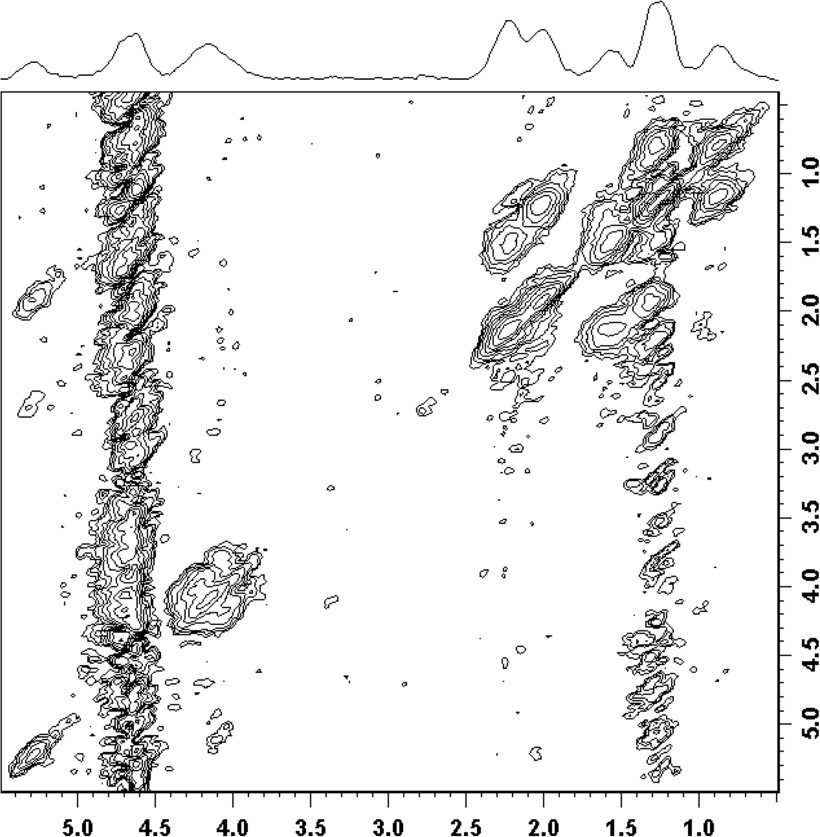

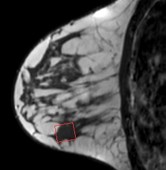


**DQF-COSY lipid composition spectrum – Tumour in patient**

F2 (ppm)

**Figure S4. Reference and polyunsaturated fatty acids (PUFA) double quantum filtered (DQF)-MRS spectra (above) and DQF – correlation spectroscopy (COSY) spectrum (below) from patients with breast cancer.**

Lipid composition spectra acquired in a pilot study in patients with invasive breast carcinoma as a consequential step of this work. The volume selections in the patients are highlighted in red. Unsuppressed reference spectrum is shown in red (5.3 ppm from both PUFA and MUFA,

2.8 ppm from PUFA), DQF-MRS PUFA spectrum is shown in blue (5.3 ppm from PUFA). The DQF-PUFA spectrum is magnified in the inset to show the clean PUFA signal peak for accurate PUFA fraction quantification. Water (4.7 ppm) and lipid resonances (0.0 – 3.0 ppm) are effectively suppressed in the PUFA spectrum. In DQF-COSY, the major diagonal peaks, including (0.9,0.9) ppm, (2.1,2.1) ppm and (4.3,4.3) ppm are well resolved. Cross peaks at (5.3,2.1) ppm (MUFA) and (5.3,2.8) ppm (PUFA) are prominent. The uncluttered spectrum facilitates accurate lipid composition quantification of the tumour in patient.

# References:

1. He Q, Shkarin P, Hooley RJ, Lannin DR, Weinreb JC, Bossuyt VIJ (2007) In vivo MR spectroscopic imaging of polyunsaturated fatty acids (PUFA) in healthy and cancerous breast tissues by selective multiple- quantum coherence transfer (Sel-MQC): A preliminary study. Magn Reson Med 58:1079–1085
2. Prescot AP, Dzik-Jurasz ASK, Leach MO, Sirohi B, Powles R, Collins DJ (2005) Localized COSY and DQF-COSY ^1^H-MRS sequences for investigating human tibial bone marrow in vivo and initial application to patients with acute leukemia. J Magn Reson Imaging 22:541–548
3. Li BS, Regal J, Gonen O (2001) SNR versus resolution in 3D ^1^H MRS of the human brain at high magnetic fields. Magn Reson Med 46:1049– 1053
4. Gruber S, Mlynárik V, Moser E (2003) High-resolution 3D proton spectroscopic imaging of the human brain at 3 T: SNR issues and application for anatomy-matched voxel sizes. Magn Reson Med 49:299– 306
5. Pradhan S, Bonekamp S, Gillen JS et al (2015) Comparison of single voxel brain MRS AT 3T and 7T using 32-channel head coils. Magn Reson Imaging 33:1013–1018
6. Zancanaro C, Nano R, Marchioro C, Sbarbati A, Boicelli A, Osculati F (1994) Magnetic resonance spectroscopy investigations of brown adipose tissue and isolated brown adipocytes. J Lipid Res 35:2191– 2199
7. Knothe G, Kenar JA (2004) Determination of the fatty acid profile by ^1^H- NMR spectroscopy. Eur J Lipid Sci Technol 106:88–96
8. Corbin IR, Furth EE, Pickup S, Siegelman ES, Delikatny EJ (2009) In vivo assessment of hepatic triglycerides in murine non-alcoholic fatty liver disease using magnetic resonance spectroscopy. Biochim Biophys Acta 1791:757–763
